# An Injectable Hydrogel as Bone Graft Material with Added Antimicrobial Properties

**DOI:** 10.1089/ten.tea.2016.0014

**Published:** 2016-06-01

**Authors:** Giacomo Tommasi, Stefano Perni, Polina Prokopovich

**Affiliations:** School of Pharmacy and Pharmaceutical Sciences, Cardiff University, Cardiff, United Kingdom.

## Abstract

Currently, the technique which provides the best chances for a successful bone graft, is the use of bone tissue from the same patient receiving it (autograft); the main limitations are the limited availability and the risks involved in removing living bone tissue, for example, explant site pain and morbidity. Allografts and xenografts may overcome these limitations; however, they increase the risk of rejection. For all these reasons the development of an artificial bone graft material is particularly important and hydrogels are a promising alternative for bone regeneration. Gels were prepared using 1,4-butanediol diacrylate as crosslinker and alpha tricalciumphosphate; ZnCl_2_ and SrCl_2_ were added to the aqueous phase. MTT results demonstrated that the addition of strontium had a beneficial effect on the osteoblast cells density on hydrogels, and zinc instead did not increase osteoblast proliferation. The amount of calcium produced by the osteoblast cells quantified through the Alizarin Red protocol revealed that both strontium and zinc positively influenced the formation of calcium; furthermore, their effect was synergistic. Rheology properties were used to mechanically characterize the hydrogels and especially the influence of crosslinker's concentration on them, showing the hydrogels presented had extremely good mechanical properties. Furthermore, the antimicrobial activity of strontium and zinc in the hydrogels against methicillin-resistant *Staphylococcus aureus* and *Staphylococcus epidermidis* was determined.

## Introduction

Bone is a rigid, mineralized connective tissue and its main function is to protect organs and give support to the body. Therefore, bones are fundamental for locomotion, but they also provide other important functions, such as regulating homeostatis of calcium and provide a source of hematopoietic stem cells.^1^ These different functions are related macroscopically to two different forms: compact (cortical) bone and trabecular (cancellous or spongy) bone. The first appears as a solid mass, represent 80% of the mass of an adult human,^2^ has a porosity of 5%,^3^ and has the function of protection and support. The trabecular bone appears as a sponge, with 90% porosity,^3^ whose free spaces are filled with bone marrow and is mainly responsible for mineral homeostasis.^4,5^

Bone matrix is composed for 77% by inorganic material (90% calcium phosphate) and for only 23% by organic material, most of which (89%) is type I collagen.^3^

Bone provides solid support to the body structure and is an organ that acts as a reservoir for critical ions such as calcium and phosphate, but it also plays an important role for the immune system; in actual fact, immune cells (T cells, B cells, and natural killer [NK] cells) are produced in the bone marrow and the interaction between immune cells and bone is classified as osteoimmunology.^6^

Unfortunately, bones are often subjected to injuries due to sports or in the workplace along with trauma commonly related to road traffic accidents^2^ and diseases such as tumors.^7,8^ The treatment of these conditions often requires the use of bone grafts in all those cases in which bone cannot self-repair and must be substituted.^9^ Today, the technique that gives the best results in terms of integration and new bone formation is the autograft (the transplant of bone tissues from the same patient).

Bone is the second most common transplanted tissue after blood, with the iliac crest autologous graft being most used.^6^ For this reason, autologous bone graft is considered the gold standard for treatment of bone defects, but several drawbacks exist.^10,11^ The main limitations of this procedure are the limited availability and the risks involved in removing living bone tissue, for example, explant site pain and morbidity.^11^ Allografts and xenografts may overcome these limitations, but they increase the risk of rejection since the host's immune system may recognize the graft as a nonself tissue^6,10^; furthermore, ethical issues surround the acceptance by a patient of having implanted tissue originated from another human being or an animal. For all these reasons the development of an artificial bone graft is particularly important and bone tissue engineering is a promising alternative for bone regeneration. Synthetic graft substitutes combine scaffolding properties with biological elements to stimulate cells proliferation and differentiation and eventually osteogenesis.^6^ They may determine a significant improvement in the restorative treatment and healthcare expenses of degenerative bone conditions, deformities, injuries, or intentional loss (e.g., cancer resection).^2^ In 2010, the sales in bone graft substitutes were valued at $1.3 billion in the United States, with a forecast of $2.3 billion in 2017.^12^ It is estimated that 2.2 million of orthopedic procedures worldwide employ bone grafts annually.^13^

During the last 15 years great effort has been dedicated to the use of hydrogels as a material for bone regeneration^12^ and cartilages.^14,15^ Hydrogels are materials that swell, but do not dissolve in water, maintaining a distinct 3D network structure by virtue of crosslinks^16,17^ and their high content of water let them mimic the composition of biological tissues. On the other hand, their mechanical properties are generally poor and cannot be applied in load-bearing situation; to date, none of the bone substitutes proposed is load bearing.^18^ Therefore, when required, the mechanical support is provided by fixation devices.^19^ Hydrogels are particularly interesting because they can be used to deliver cells, drugs, or proteins. In fact, several different combinations of hydrogels and additives have been studied and many of them demonstrated promising results.^20–24^ However, the main reasoning for research, focusing on hydrogels for bone regeneration, is their injectability.^1^ The main advantage of injectable materials compared to 3D scaffold is that they can be applied using noninvasive or minimally invasive surgeries and they can fill irregularly shaped bone defects easily. Therefore, using an injectable hydrogel, instead of an autologous bone graft, has a double advantage: the primary surgery to implant the gel would be noninvasive and the secondary surgery to explant the graft would be avoided. In the development of injectable hydrogels, two main classes of materials have been used, natural and synthetic polymers.^1^ Hyaluronate-^25,26^ and chitosan-^27^ based hydrogel are examples of the former type, whereas PEG-^28–30^ and PLGA-^31^ based hydrogels belong to the latter type.

We have previously developed synthetic bone graft PEG-based hydrogels using acrylate monomers [PEGMEM (poly(ethylene glycol) methyl ether methacrylate), DEM (2-(dimethylamino)ethyl methacrylate)] crosslinked with N-N′-methylenebisacrylamide^32,33^ that exhibited high mechanical properties, whereas calcium phosphate particles were formed inside the gel matrix, postgelification, using a reaction–diffusion method. In this work, we developed and *in vitro* tested a new acrylate-based hydrogels adding calcium phosphate directly in the gelifying solution and such not requiring the additional step of the reaction–diffusion process. Furthermore, injectability was conferred by the use of a diacrylate ester as crosslinker instead of N-N′-methylenebisacrylamide^32,33^ that requires the removal of unreacted molecules through time-consuming dialysis. We also added Zn and Sr to the hydrogels as they are known to enhance osteoblast growth^34^ and demonstrate that these ions also confer antimicrobial properties to the material.

## Materials and Methods

### Materials

PEGMEM [poly(ethylene glycol) methyl ether methacrylate] with average Mn = 300, DEM [2-(dimethylamino)ethyl methacrylate], 1,4-butanediol dimethacrylate, ammonium persulfate (APS), ZnCl_2_, SrCl_2,_ glutaraldehyde, acetic acid, Thiazolyl Blue Tetrazolium Bromide (MTT), and Alizarin Red S were purchased from Sigma-Aldrich. α-Tricalcium-phosphate (α-TCP) was purchased from Fluka.

Simulated body fluid (SBF) solution was prepared according to British Standard ISO 23317:200.

Gram-positive bacteria, methicillin-resistant *Staphylococcus aureus* (MRSA)—MRSA (NCTC 12493) and *Staphylococcus epidermidis* (RP62a), were used. Bacteria frozen stocks were stored at −80°C; weekly, strains were streaked out on Brain Heart Infusion (BHI) plates (Oxoid) and incubated for 24 h at 37°C and then stored at 4°C.

### Hydrogel preparation

Hydrogel samples were prepared mixing 2 mL of PEGMEM, 1 mL of DEM, and 1,4-butanediol diacrylate as crosslinker in different concentrations: 1%, 5%, 10%, 20% (v/v) in 5 mL of deionized water containing 2 mg/mL of APS and 10 mg/mL of α-TCP. When required, ZnCl_2_ and SrCl_2_ were added to the aqueous phase at various concentrations (100 or 50 mM for SrCl_2_ and 0.5 or 1 mM for ZnCl_2_), individually or together. Gels were prepared in 10-mL plastic syringes and, once polymerized, the top of syringes were cut and gels were pushed out from the syringe shaft using the plunger.

### Physicochemical characterization of hydrogels

#### Temperature profile during polymerization

The polymerization stage is an exothermic reaction. It means that during polymerization, energy is released and the material increases its temperature. To quantify this increment, the temperature has been monitored during gelification through the insertion of a thermocouple in the gel. Gels were prepared directly inside 50-mL plastic falcon tubes, in which the thermocouple (EKT 3001; Heidolph) was inserted carefully preventing touching the tube walls. Temperature was recorded every minute for 40 min since mixing of the monomers with the water solution containing APS. For this test, three independent gels with 1% and 20% v/v crosslinking agent were prepared.

#### Swelling test

Hydrogels after polymerization were then cut into smaller pieces and put in glass bottles containing phosphate-buffered solution (PBS) and incubated at 37°C. Gels were weighted before the immersion in 5 mL of PBS and at different time points: every day for the first 21 days, twice a week for the next 50 days, and once a week afterward. At least three individual samples were weighted and averaged for each condition.

The swelling ratio *(H)* was calculated as a percentage variation of the initial weight according to:

\begin{align*}H = \frac { m_f }  { m_i } \times 100 \tag { 1 } \end{align*}

where: *m_i_* is weight of the gel before swelling, *m_f_* is weight of the gel after swelling.

Freshly made samples were employed to avoid the possibility of drying when in contact with the atmosphere. The test was performed on three independently prepared samples and results are presented as mean ± standard deviation (SD).

#### Rheology

Rheology tests were performed to characterize the time required to achieve gelification and the mechanical properties of gels, especially the influence of crosslinker's concentration.

The time required by the hydrogel to form was determined as the time needed by the phase angle to drop to zero, and for this, an aliquot of about 100 μL of the suspension before gelification started was put in the rheometer (AR-G2; TA Instruments) plate and time sweep mode was used at a frequency of 1 Hz for 1200 s period.

Hydrogels were stored in PBS for 14 days at 37°C and then they were cut into a circle through a stamp of 25 mm diameter and loaded into the rheometer; the frequency sweep mode (from 10 to 0.01 Hz) was employed for the determination of the rheological properties of the hydrogels.

All tests were performed at a controlled temperature of 37°C with a 25-mm serrated parallel plate (Peltier plate made of aluminum) to prevent slippage sandwiching the material at a constant, but low normal force (5 N); measurements were conducted at a strain of 0.1%, which was within the linear viscoelastic range of the material, as confirmed by a strain sweep and the absence of a third harmonic response.

#### X-ray diffraction

Samples prepared as for swelling test remained immersed in SBF at 37°C for 1 month. Before analysis, samples were removed from the SBF and freeze dried for 24 h at −40°C using an Edwards Modulyo Freeze Dryer. X-ray diffraction (XRD) patterns were acquired using a Philips PW1710 Automated Powder Diffractometer and samples were run over a 2θ range between 10 and 60° at a scan speed of 0.016°/s.

#### Scanning electron microscope

Samples of gels freshly prepared and after 1 month in SBF were analyzed with scanning electron microscope (SEM) (Gemini, Zeiss 1540xb; Oxford Instruments). Before the analysis all samples were freeze dried for 24 h. The applied voltage used was between 3 and 5 kV; the range of magnifications used was from a minimum of 40 × to a maximum of 10,000 ×.

#### Ion release

Ion release test was performed to determine the concentration of Sr and Zn ions released from the gels with 1% and 20% v/v of crosslinker, with 50 or 100 mM Sr and a combination of 100 mM of Sr and 1 mM of Zn. Gels were prepared in plastic syringes and after gelification were cut in smaller pieces and immersed in 5 mL of PBS inside glass bottles. Bottles were incubated at 37°C. For every time point, samples were transferred in 5 mL of fresh PBS, while the whole solution in which they were immersed was diluted at a ratio of 4:1 with PBS and analyzed with spectrometer (Optima 2100 DV; PerkinElmer). Calibration was performed with 28-element Standard (Fisher Scientific); strontium was detected with 407.771 nm filter, whereas zinc was detected with 206.200 nm filter. The test was performed on three independently prepared samples and results are presented as mean ± SD.

### Osteoblast cell growth on hydrogels

Osteoblast cells (MC-3T3) were cultured in Dulbecco's modified Eagle's medium (DMEM) supplemented with fetal bovine serum (10% v/v) and 1% v/v of a solution of penicillin (5000 U/mL)/streptomycin (5000 mg/mL); cells were incubated at 37°C in humidified atmosphere with 5% CO_2_. Cells were grown till ∼70% confluence, washed twice with sterile PBS, and detached with trypsin; osteoblast cells were counted (using Trypan Blue to differentiate between viable and nonviable cells) and diluted to a concentration ∼10^5^ cells/mL with fresh medium.

Gels were prepared directly in round-bottom 96-well plates, for a total volume of 100 μL of gel per well and stored at room temperature for 24 h before cells were put in contact with. Each well was filled with 100 μL of cell suspension containing about 10,000 mouse osteoblast (MC-3T3) cells in DMEM medium. Plates were incubated at 37°C in humidified atmosphere with 5% CO_2_; medium was regularly changed for 21 days when MTT and Alizarin Red tests were carried out. The test was performed in triplicates on three independently prepared samples and results are presented as mean ± SD.

#### Thiazolyl Blue Tetrazolium Bromide (MTT)

The medium present in the well was taken off and replaced with 100 μL of fresh medium (phenol red-free). Ten microliters of Thiazolyl Blue Tetrazolium Bromide 5 mg/mL in PBS was added to each well and the plate was incubated for 2 h at 37°C in humidified atmosphere with 5% CO_2_; after this 50 μL of dimethyl sulfoxide (DMSO) was added and the plates were incubated for further 10 min. Eighty microliters of solution containing the dissolved formazan was put in another 96-well plate and analyzed with a spectrophotometer (LabTech LT5000MS) at 560 nm.

#### Alizarin Red

The medium present in the well was taken off and replaced with 100 μL of glutaraldehyde 10% (v/v); the plates were incubated for 15 min and washed with deionized water. One hundred microliters of Alizarin Red S 1% (v/v) was added to each well and the plates were incubated for 20 min. After washing with deionized water, 150 μL of acetic acid 10% (v/v) was added to each well and the plates were incubated for 30 min. After this, 20 μL of solution was put in another 96-well plate, diluted with 80 μL of deionized water, and analyzed with a spectrophotometer (LabTech LT5000MS) at 450 nm.

Controls samples consisting of unseeded gels were used as control for Alizarin Red test because of the presence of calcium in the hydrogels through α-TCP.

### Antimicrobial activity of hydrogels

Gels were prepared directly inside a 24-well plate and for each well a total of 1 mL of gel was used. Once the gels were formed, 1.5 mL of bacterial broth suspension, obtained by incubating statically 10 mL of fresh sterile BHI broth (Oxoid) for 24 h at 37°C after inoculating with a loopful of cells, was put into each well. Bacteria were left in contact with the gels in the incubator at 37°C for 1 h. After incubation, the suspension was removed and the gel was washed three times with 1 mL of sterile PBS. Wells were then filled with 1.5 mL of solution composed by 10% of sterile BHI broth and 90% of sterile PBS and finally incubated at 37°C. After 24 h, 50 μL of suspension from each well was mixed with 100 μL of fresh BHI broth placed in 100-well plates and growth curves at 37°C were recorded and analyzed using Bioscreen plate reader. Optical density at 600 nm of each individual well was recorded every 15 min for a total of 24 h.

Tests were performed in triplicate from three independent cultures for a total of nine growth curves for each bacterium on each gel. Each growth curve was fitted using the Gompertz growth model to extract values of lag phase and growth rate. Results are presented as mean and standard deviation.

### Statistical analysis

Data were analyzed using one-way ANOVA to determine any significant difference between the mean values, and this was followed by Tukey's *post hoc* test (*p* < 0.05). Statistical analysis was performed using SPSS.

## Results

### Temperature profile during polymerization

**FIG. 1.**
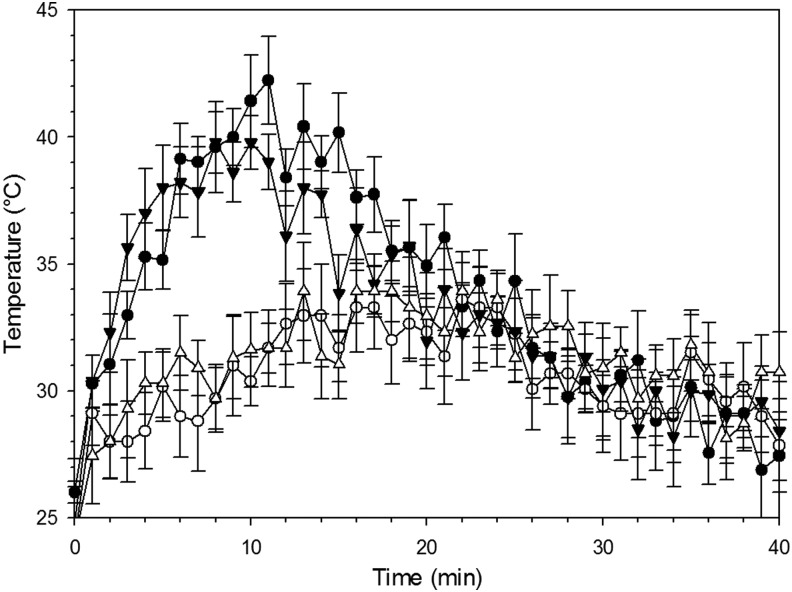
Temperature profile during polymerization as function of crosslinker concentration and presence of Sr ions. ● 1% ◯ 20% ▼ 1% + Sr 100 mM △ 20% + Sr 100 mM

The temperature profile during polymerization of the hydrogels showed that gels with 1% of crosslinker had a higher temperature increment than those with 20%, during polymerization (Fig. 1). Similarly, the presence of strontium in both gels, with 1% and 20% of crosslinker, did not change the temperature profile significantly. The monomers and crosslinker were mixed at room temperature, and temperature rose in the first 10 min as a result of the exothermic polymerization reaction, and later the temperature gradually decreased.

### Polymerization time

**FIG. 2.**
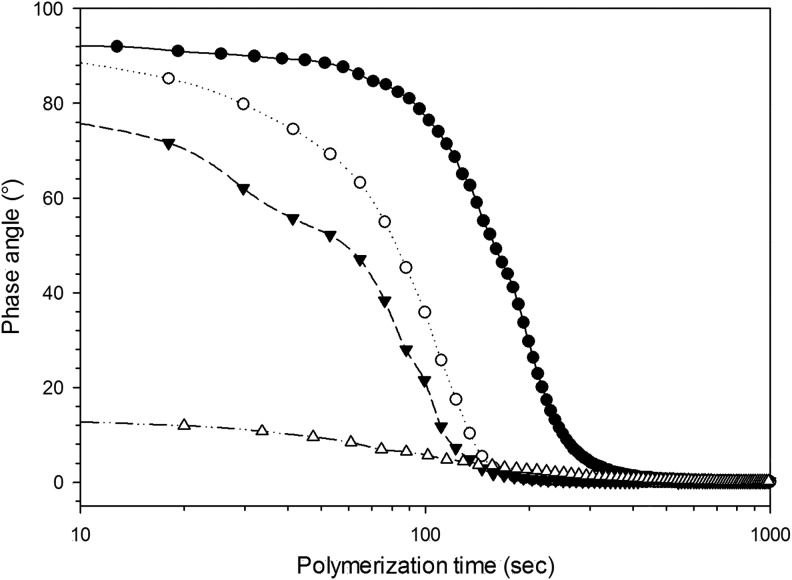
Variation of phase angle as function of crosslinker concentration during polymerization. ● 1% ◯ 5% ▼ 10% △ 20%

The time needed to form the gel was determined from rheological tests of phase angle in time sweep mode; when the phase angle between storage and loss modulus reaches a value close to 0° the material was completely gelled. In Figure 2 it can be noticed that every gel reached a phase angle of about 0° in 100–300 s, this time slightly decreased with increasing content of crosslinker. For gels with >10% v/v of crosslinking agents, the phase angle was very high at the point of mixing and decreased with advancing polymerization.

### Swelling

**FIG. 3.**
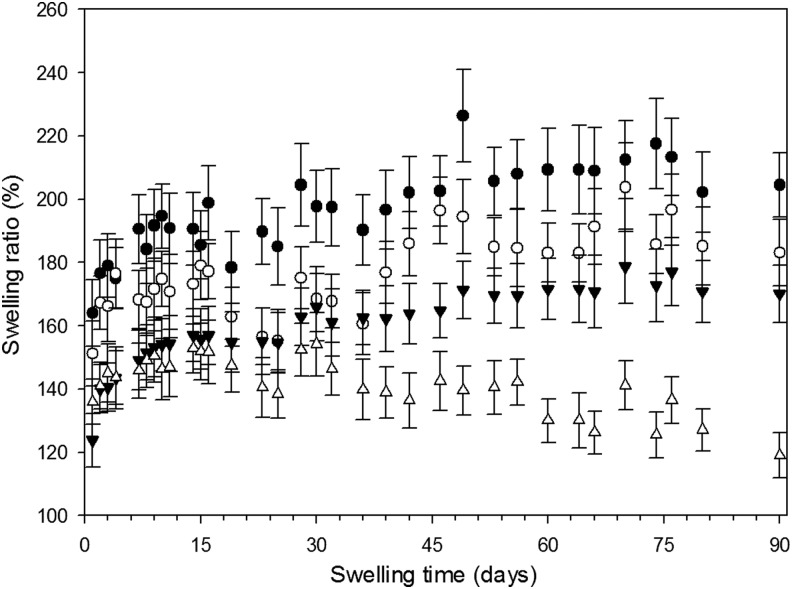
Influence of crosslinker concentration on water adsorption of hydrogels over time. ● 1% ◯ 5% ▼ 10% △ 20%

As can be seen from the graphs (Fig. 3), water uptake for all hydrogels reached a maximum after about 10 days of immersion; as expected, the lower the concentration of crosslinker, the higher the swelling. When 1% v/v crosslinker was used, the gels almost doubled their weight, whereas the gels containing 20% v/v of crosslinking agent increased their weight of 50%. After the first 2 weeks the weight of the samples remained constant for the remaining duration of the test (up to 90 days) for gels with crosslinker concentration <10% v/v; gels prepared with 20% v/v exhibited a weight drop probably in virtue of the hydrolysis of the ester bonds present in the crosslinking molecule that resulted in the acrylate chains being released from the solid gel.

### Rheology

**FIG. 4.**
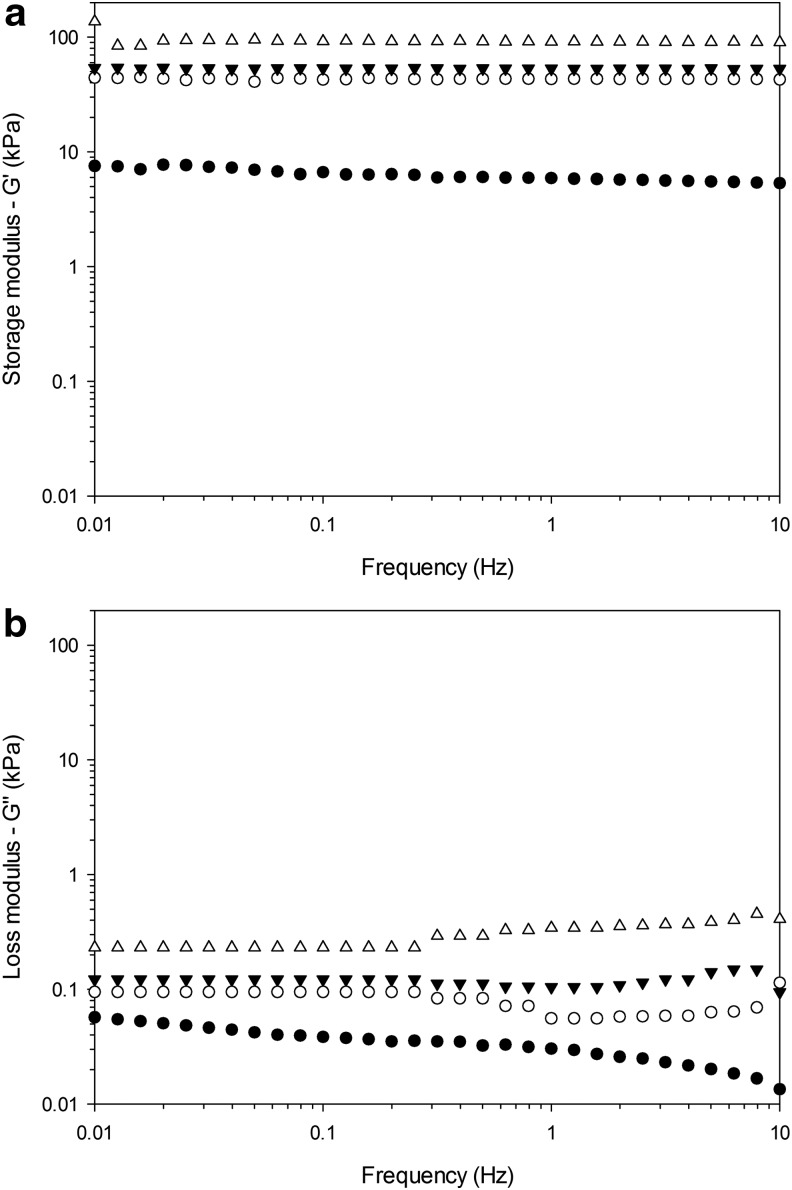
Frequency dependence of storage modulus *G*′ **(a)** and loss modulus *G*″ **(b)** of hydrogels with different concentrations of crosslinker. ● 1% ◯ 5% ▼ 10% △ 20%

Storage modulus (*G*′) results indicated that mechanical properties were directly proportional to the concentration of crosslinker, as expected, because the more the interchain bonds, the greater the force required to separate them (Fig. 4). The highest value of *G*′ was about 100 kPa and the lowest 8 kPa.

As concerns the loss modulus (*G*″), the values obtained are significantly lower than storage modulii and were also directly correlated to the concentration of crosslinker. The highest value in *G*" corresponded to 20% v/v and was about 0.1 kPa, about three orders of magnitude lower than its storage modulus.

Both storage and loss modulii were independent of the frequency of the applied load, thus indicating a strong elastic behavior of the gels.

No effect was noticed when Sr and Zn, individually or together, were added to the hydrogels compared with the samples without added salts (data not shown).

### Scanning electron microscope

**FIG. 5.**
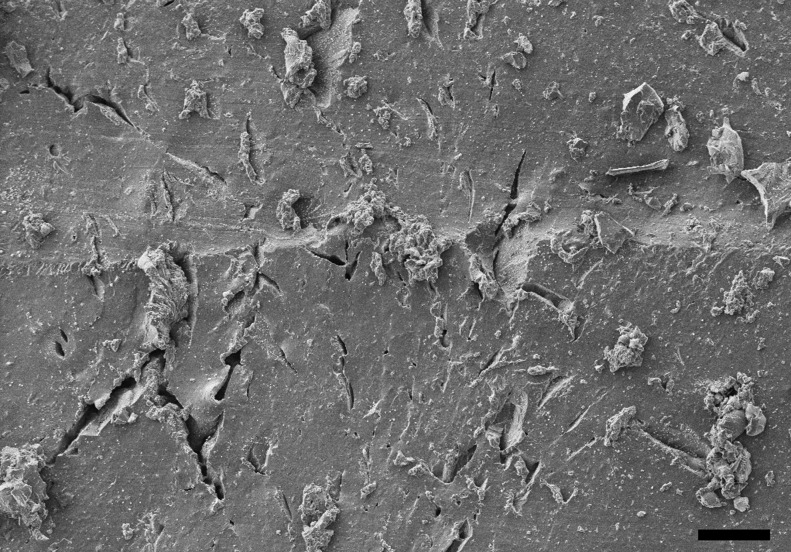
Example of SEM image of the hydrogel surface after immersion in SBF for after 1 month. Bar represent 100 μm. SEM, scanning electron microscope.

Deposits were visible with naked eyes on hydrogel samples, which originally were transparent, after 1 month in SBF. This phenomenon is appreciable in the SEM picture (Fig. 5), where the formation of crystals is clearly noticeable.

### X-ray diffraction

**FIG. 6.**
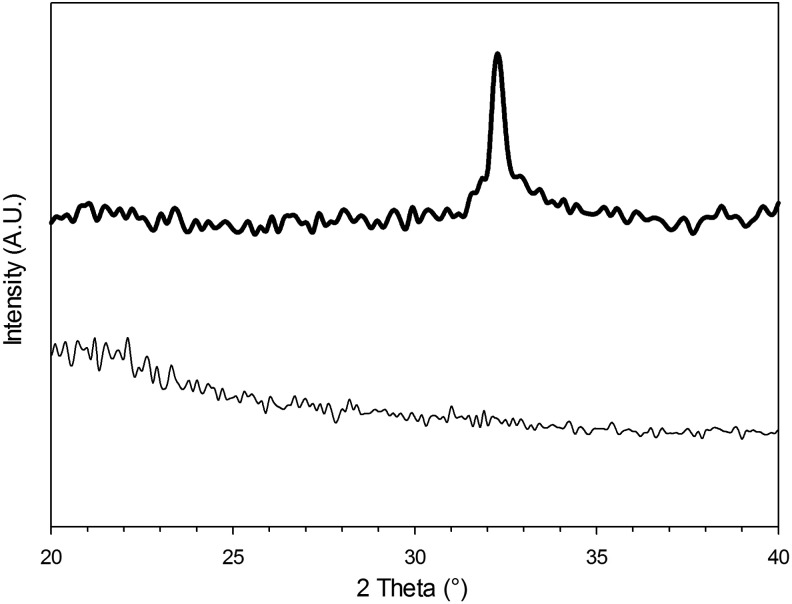
XRD patterns of hydrogels with 20% v/v crosslinker. XRD, X-ray diffraction. ____ after 0 h 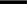 after 1 month in SBF

XRD pattern of pure hydroxyapatite exhibits three high-intensity peaks located at 2θ = 31.7°, 32.2°, and 32.9°.^35^ The confirmation that the deposits observed on the surface of the gels, after immersion in SBF, were hydroxyapatite was provided by XRD pattern of the gels that revealed the presence of peaks at about 2θ = 32° (Fig. 6). This was not dependent on either the crosslinker concentration in the hydrogels or the presence of Sr and Zn.

### Ion release

**FIG. 7.**
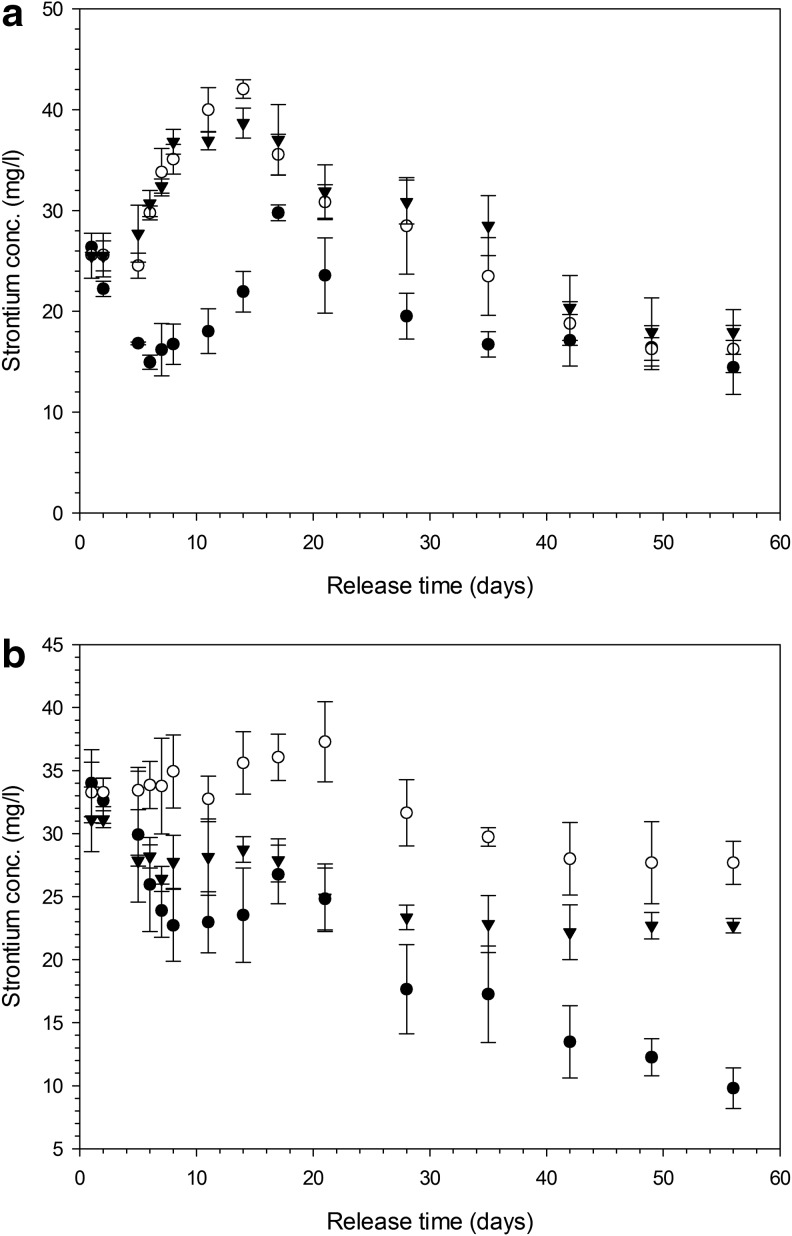
Concentration of Sr released from hydrogels with 1% **(a)** and 20% **(b)** crosslinking concentration. ● 50 mM Sr ◯ Sr 100 mM ▼ Sr 100 mM + Zn 1 mM

The concentration of Sr in the fluid in contact with hydrogels containing 1% v/v crosslinker and 100 mM of SrCl_2_ in the formulation increased during the first 10 days (Fig. 7a), irrespectively of the presence of ZnCl_2_. Gels synthesized with 50 mM of Sr in the first 2–3 days exhibited the same Sr release as those prepared with 100 mM, but then showed a decrease and a further maximum after about 10 days (Fig. 7a); all gels kept releasing Sr for at least 60 days. Hydrogels prepared with 20% v/v crosslinker had a higher initial Sr release than those formulated with 1% v/v crosslinker (Fig. 7b) and the Sr concentration gradually decreased after the first contact with fluids. When 50 mM was added to the hydrogel composition, the Sr concentration was lower than when 100 mM was added (Fig. 7b).

**FIG. 8.**
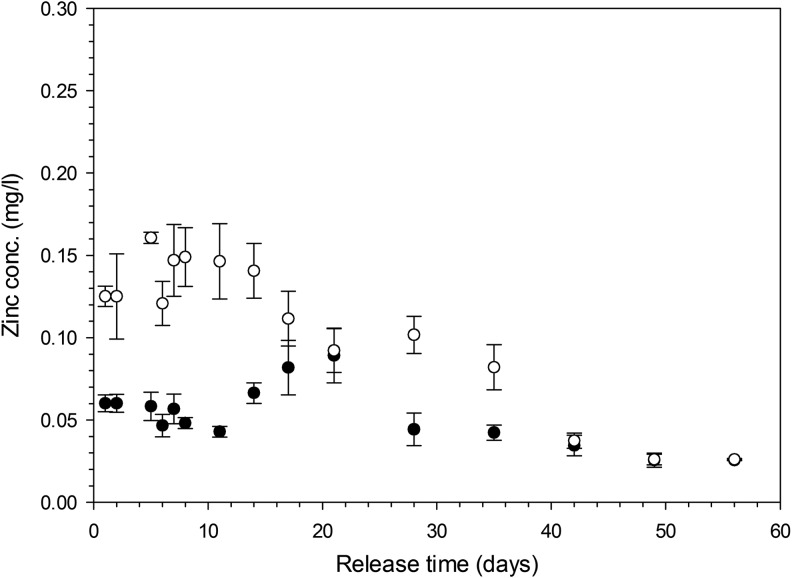
Concentration of Zn released from hydrogels with 1% (●) and 20% (◯) crosslinking concentration containing ZnCl_2_ 1 mM.

The release of Zn from the gels exhibited a maximum after about 15–20 days for hydrogels made with 20% v/v crosslinker and after about 2–3 days for hydrogels made with 1% v/v crosslinker (Fig. 8); in both cases Zn was recorded for the duration of the experiment with concentrations gradually decreasing after the mentioned relative maximum.

### Thiazolyl blue tetrazolium bromide (MTT)

**FIG. 9.**
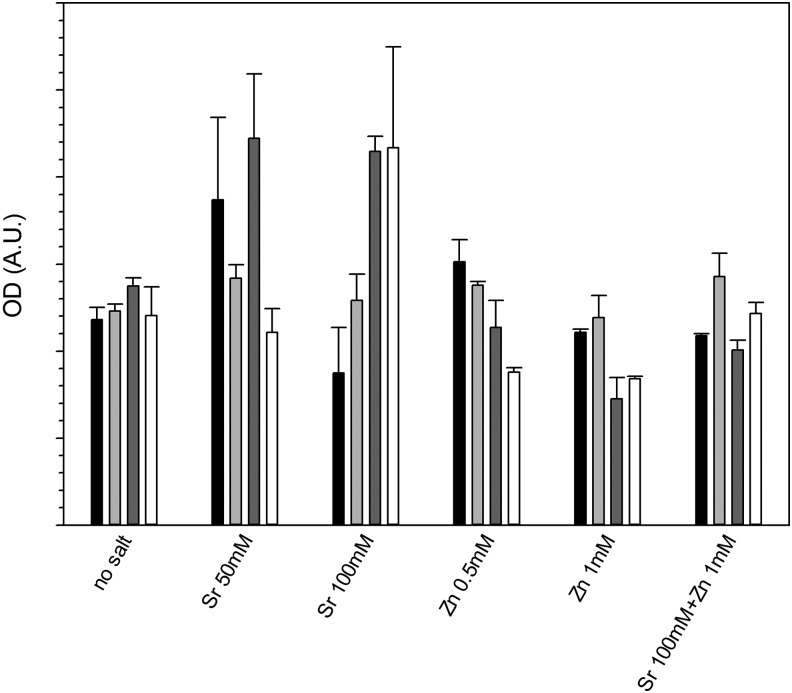
MTT assay on osteoblast cells grown 21 days on hydrogels containing combinations of Sr and Zn. 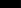
 1% 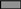 5% 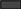 10% 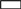 20%

The viability of the osteoblast cells seeded on the hydrogels was assessed with the MTT assay (Fig. 9) that revealed the level of crosslinker did not influence the viability of cells when Sr or Zn was added (*p* > 0.05). When 50 mM of SrCl_2_ was added, no significant difference was noticed across the concentration of crosslinker (*p* > 0.05), whereas with 100 mM of SrCl_2_ higher metabolic activity was recorded for gels prepared with 10% and 20% v/v of crosslinker (*p* < 0.05). ZnCl_2_ did not return significant difference from samples without this salt (*p* > 0.05).

### Alizarin Red

**FIG. 10.**
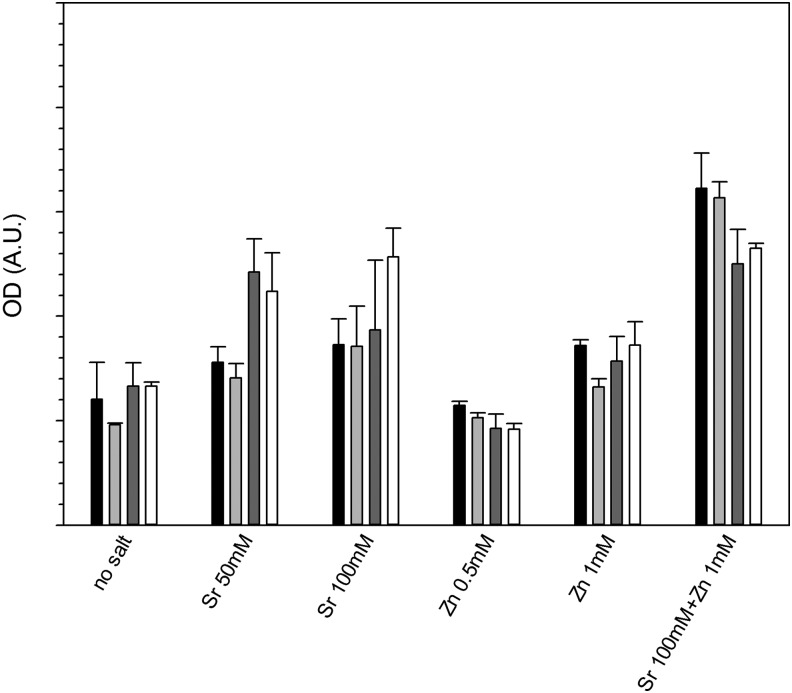
Alizarin Red assay on osteoblast cells grown 21 days on hydrogels containing combinations of Sr and Zn. 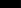
 1% 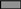 5% 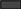 10% 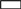 20%

The calcium formation resulting from the growth of osteoblast cells on the hydrogels was assessed through Alizarin Red staining (Fig. 10); the presence of Sr resulted in an increase of calcium formation on gels containing 10% and 20% v/v crosslinker, also Zn did not appear to significantly affect the results (*p* > 0.05).

### Antimicrobial test

**FIG. 11.**
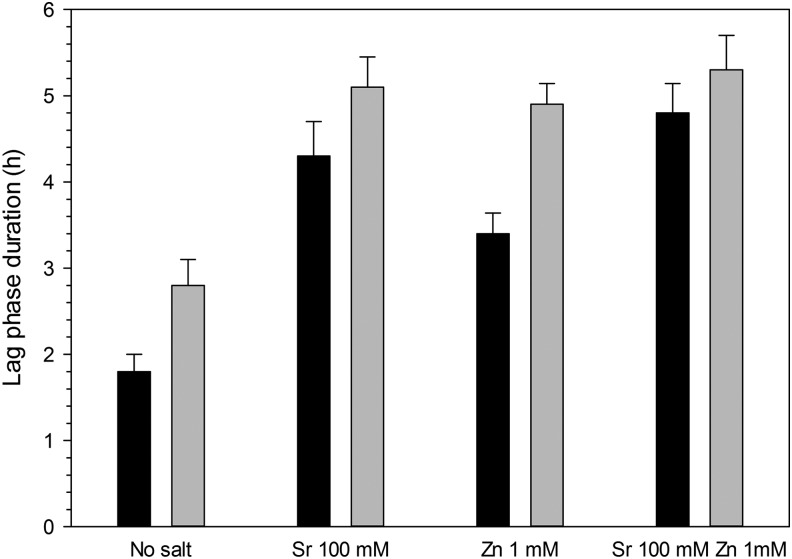
Apparent Lag phase duration of bacteria grown on hydrogels containing 20% v/v of crosslinker and SrCl_2_ and ZnCl_2_. MRSA, methicillin-resistant *Staphylococcus aureus*. 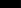
 MRSA 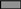 *S. epidermidis*

The antimicrobial activity of the most promising hydrogels (with 20% v/v of 1,4-butanediol diacrylate), as seen before, was tested against *S. epidermidis* and MRSA. Both bacteria tested are common sources of postorthopedic surgeries^36–38^; practically, no antimicrobial activity was exhibited by hydrogels not containing either Sr or Zn (Fig. 11) as the apparent lag phase is typical of cell suspensions with high initial concentration.^36,39,40^ However, when Sr was added to the hydrogel formulation, both bacteria displayed lag phases about 2 h longer; this difference was statistically significant (*p* < 0.05) and corresponded to a reduction >90% (1 log_10_) of the bacteria. The addition of Zn, on the other hand, had a similar effect on *S. epidermidis*, but smaller on MRSA; moreover, no synergistic effect between Sr and Zn was detected as apparent lag phase durations were not statistically different in case of Sr alone or Zn alone (*p* < 0.05).

## Discussion

### Hydrogels preparation

Injectability, for a hydrogel, requires all reagents employed in the synthesis to be biologically safe because of the unreacted molecules possible release from the matrix subsequent to the implantation. In our previous works, we have developed other hydrogels^32,33^ with the use of N-N′-methylenebisacrylamide as a crosslinking agent; this resulted in the need for dialysis of the prepared hydrogels because of the adverse effects caused by such molecule; hence the hydrogels made with this crosslinking agent were not injectable. In this study, we replaced N-N′-methylenebisacrylamide with 1,4-butanediol diacrylate, the latter is used in dental resins^41^ and does not require dialysis of the hydrogels while still providing the same crosslinking function, therefore, providing the chemical safety property necessary to be injectable. To establish the feasibility of the developed hydrogels for injectable bone graft applications other properties (gelification time and temperature) that are essential to such characteristic were also investigated. The gelification needs to occur in a time allowing manipulation and implantation while the temperature during the gelification process must not reach levels causing tissue damage. A higher concentration of crosslinker appeared to reduce the setting time (Fig. 1) as expected because a 3D network between chains is established quicker with increasing ratios crosslinker to monomers; at the same time, the temperature rise during gelification was reduced with increasing concentration of crosslinker (Fig. 2). This was a consequence of the monomers/radicals reduced mobility and probability of reaction caused by the quicker hydrogel hardening compared with the case of lower crosslinker concentration. In the worst case (1% v/v of crosslinker), the temperature reached 42°C starting from 25°C. This increment, which is still significantly lower than the maximum temperature reached by bone cement during setting, can still be considered acceptable for an eventual application in bone tissue engineering as it is unlikely causing damages to the surrounding tissue, and drugs could also be incorporated into the gel without thermal degradation. In fact, gels could be injected at 20°C reaching 36°C, assuming the same ΔT. As concerns the time required to return to initial temperature, it must be considered that during this experiment the samples were kept in a plastic tube, surrounded by air; whereas in an *in vivo* application, gels would be surrounded by tissues at 37°C and this would help them reach body temperature and remain at it in consequence of the better heat transfer properties of body fluids compared with air. From an application point of view, the gelling time of the hydrogels presented here is good. In fact, a shorter time, like 1 min, could be difficult to handle as gels could harden before being properly applied to the site of interest; on the other hand, long times are not appropriate either as gels could be injected, but move from the wanted site. In 300 s the gel could be mixed in a syringe and injected before completely gelled; for comparison, PMMA bone cement is mixed and applied in about 2–3 min.

Hydrogels have the capacity of absorbing water when exposed to an aqueous phase, this capacity is linked to the flexibility of the gel matrix and the amount of water employed during the initial formulation.

Similar hydrogels prepared using N-N′-methylenebisacrylamide as crosslinking agent returned a lower water uptake.^32^ In this work, we used 1,4-butanediol diacrylate that presents a longer chain between the two acrylate groups than N-N′-methylenebisacrylamide and this is likely to provide more flexibility and water adsorption capacity to the hydrogels presented in this work. Moreover, the presence of ester instead of amide bonds allows biodegradation of the biomaterial. The degradation of the hydrogel is a potentially welcome property as long as the dissolution is not quicker than the bone formation in the voided space, in this case mechanical weakening could result in graft failure. The hydrogels developed in this work does not exhibit a concerning kinetic of hydrolysis as seen in Figure 3.

Currently, one of the main limitations to the use of hydrogels in orthopedic applications is represented by the weak mechanical properties generally exhibited by hydrogels; this limits many applications to no load-bearing locations.^42^ The values of *G*′ (about 0.1 MPa) obtained for the hydrogels developed in this work (Fig. 4) were encouragingly high for hydrogels. They exhibited similar rheological properties to PMMA bone cement^36^ or to mineralized acrylate-based hydrogels through reaction–diffusion methods resulting in the formation of beta-TCP inside the hydrogel matrix.^33^ Hence, they are likely to be suited for implantation in load-bearing sites.

### Growth of osteoblasts on hydrogels

Assessment of apatite formation on a biomaterial following exposure to SBF solution is the standard method to determine the capacity of a material to stimulate bone formation.^43^ We observed the formation of crystals on hydrogel surface through SEM (Fig. 5) and assessed them to be hydroxyapatite using XRD (Fig. 6), thus providing conclusive evidence of the suitability of the hydrogels presented in this work to be employed as bone graft material.

Bones are composite materials that are predominantly made of collagen and hydroxyapatite.^44^ α-TCP is sometimes added to synthetic bone graft materials^45,46^ as it is biocompatible, improves cell proliferation,^45^ and hydrolyses to hydroxyapatite,^47^ thus inducing the biomaterial to simulate the mineralogical composition of bone. Beta-TCP has also been added to bone graft material^48,49^; we chose α-TCP as it has higher solubility than beta-TCP, hence a quicker degradation.^50^ The concentration use in this work (0.8% w/w) was similar to other works employing calcium phosphate in hydrogels for bone replacement^49^ and an order of magnitude lower than Li *et al.*^45^

Strontium^34,51–55^ and zinc^56^ are known to support osteoblast growth and have been added to hydrogels developed for orthopedic applications,^34^ on the surface of bioglasses,^57,58^ on titanium surfaces,^59^ or calcium phosphate films.^60^ Release of these ions from the solid matrix is also required to stimulate osteoblast growth. The maximum concentrations of SrCl_2_ and ZnCl_2_ added to the gelification mixture were chosen in the same range of those used^34^ to crosslink an alginate base hydrogel and resulted in concentrations of ions in the release media after the first few days in the same order of magnitude. The release of these ions from the hydrogels did not follow Fick's law (Figs. 7 and 8) as the swelling of the gels is also involved in the mass transfer of ions from the solid matrix of the gel to the surrounding fluid; this is particularly evident in the gels containing 1% crosslinker, where a maximum concentration of Sr was recorded after about 10 days and of Zn after 15–20 days of contact with fluid. This phenomenon is a consequence of these gels swelling more than those containing 20% v/v crosslinker (Fig. 3); also the concentration of ions in the gels depleted from the level of the initial synthesis (*t* = 0) and as such, the concentration of ions (both Sr and Zn) in the fluid, during the first days of contact, is lower for the hydrogels with lower levels of crosslinker. In our hydrogels, Sr provided the expected activity toward bone forming cells (Figs. 9 and 10), while Zn did not. The different concentrations of these two ions in the medium after release can account for such result.

Hydrogels prepared with 20% v/v of crosslinker with Sr and Zn appeared to be the most effective as they returned the best mechanical properties and osteoblast growth and, therefore, they were selected for further investigation.

To improve cell adhesion on orthopedic materials, RGD (arginine-glycine-aspartic acid) sequences can be grafted to the surface^61^ or conjugated to hydrogel constituents.^34^ However, these do not appear essential as osteoblast adhesion has been observed on surfaces not exhibiting such features like those presented in this work and others i.e., PMMA bone cements. We chose not to pursue this approach because of the increased complexity (measurable in term product unit fabrication cost) induced by additional preparation as the incorporation of TCP in the hydrogel is likely to play a role in facilitating osteoblast adhesion and proliferation.^48,62,63^

### Antimicrobial properties

Strontium is known to interfere with bacterial growth and has been used as a possible strategy in the fight against orthopedic infections^64,65^; therefore, we hypothesized that the hydrogels containing these ions could also exhibit the capacity of interfering with bacterial infections. We employed the indirect method developed by Bechert *et al.*^66^ that is based on the duration of the apparent lag phase of the cells grown on the material measured through turbidity. The lower the cell concentration on the sample in contact with the material, the longer the apparent lag phase (time required for the cell to grow to the threshold concentration of detection through turbidity); furthermore, assuming a doubling time of 30 min, an increment in apparent lag phase of 1.5 h is equivalent to a reduction of cell concentration of 1 log_10_ because log_2_10 = 3.3.^36^ Evidence of lethal activity against pathogenic bacteria was observed (Fig. 11). The different response of these two species related to the intrinsic susceptibility of such bacteria and expected as MRSA is usually more resistant than *S. epidermidis* to antimicrobial agents.^36,39,40^

However, although the antimicrobial activity is not comparable to antibiotics or silver,^36,39,40^ the presence of Sr and Zn could be helpful in lowering the risk of bacterial infections that are always a possible threat after surgery. Because of the nonantibiotic nature of the antimicrobial compounds added to the hydrogels (Sr and Zn), this approach is also well in line with the effort against the growing concern posed by rising antibiotic resistance among pathogens.

## Conclusions

The novel hydrogels presented showed very interesting properties. First of all the mechanical properties of the hydrogels are unique, with elevated storage modulus and low viscous modulus, which can be helpful for bone tissue applications in load-bearing situations currently precluded to many hydrogels. Temperature increase during polymerization is also acceptable. Even more, the addition of strontium and zinc not only improved the growth and calcium production of osteoblast cells, but also hampered the growth of infection causing bacteria like *S. epidermidis* and MRSA.

Another important quality of the gel is the capability of forming hydroxyapatite deposits, critical in the developing process of the mineralized extracellular matrix in bone tissue applications, already after 1 month. The joint actions of ions supporting osteoblast growth (Sr and Zn) and the capability of forming hydroxyapatite deposits, due to the gel itself, can lead to a better and quicker formation of extracellular matrix.
